# Quantification of plasma cell dynamics using mathematical modelling

**DOI:** 10.1098/rsos.170759

**Published:** 2018-01-24

**Authors:** Marcel Mohr, Dirk Hose, Anja Seckinger, Anna Marciniak-Czochra

**Affiliations:** 1 Heidelberg University, Institute of Applied Mathematics, BIOQUANT and IWR, Heidelberg, Germany; 2 Heidelberg University Hospital, Medical Clinic V, Heidelberg, Germany

**Keywords:** plasma cell, bone marrow niche, vaccination, cell population dynamics, mathematical model

## Abstract

Plasma cells (PCs) are the main antibody-producing cells in humans. They are long-lived so that specific antibodies against either pathogens or vaccines are produced for decades. PC longevity is attributed to specific areas within the bone marrow micro-environment, the so-called ‘niche’, providing the cells with required growth and survival factors. With antigen encounters, e.g. infection or vaccination, new PCs are generated and home to the bone marrow where they compete with resident PCs for the niche. We propose a parametrized mathematical model describing healthy PC dynamics in the bone marrow. The model accounts for competition for the niche between newly produced PCs owing to vaccination and resident PCs. Mathematical analysis and numerical simulations of the model allow explanation of the recovery of PC homoeostasis after a vaccine-induced perturbation, and the fraction of vaccine-specific PCs inside the niche. The model enables quantification of the niche-related dynamics of PCs, i.e. the duration of PC transition into the niche and the impact of different rates for PC transitions into and out of the niche on the observed cell dynamics. Ultimately, it provides a potential basis for further investigations in health and disease.

## 1. Introduction

A plasma cell (PC) is a differentiated B cell, i.e. a type of a white blood cell which secretes antibodies. Antibodies, also referred to as immunoglobulins (Ig), are proteins which neutralize pathogens such as bacteria or viruses. PCs are mainly located in the bone marrow, where they may be long-lived, producing antibodies against pathogens for decades after the first antigen contact [1–5]. We refer the reader to [6] for a recent review of PC generation. Persistence of specific Ig is owing to the longevity of the respective PC population [7,4]. PCs are in a tight bidirectional interaction with a variety of cellular populations of the bone marrow micro-environment, e.g. stromal cells, endothelial cells, osteoclasts, as well as the extracellular matrix [8–15]. The micro-environment provides the cells with the needed growth and survival factors [2,16–20]. Respective spaces in which PCs are located are as a whole termed as ‘niche’. Since PCs die *ex vivo* within a few days in culture, they are intrinsically short-lived, and their survival depends on and is regulated by the ability to access a niche [4,10,21–24].

Vaccination denotes the administration of antigenic material, i.e. a vaccine to stimulate an immune reaction. In general, a physiological immune reaction leads, after antigen exposure, to a clonal expansion of highly proliferative antigen-specific plasmablasts (PBs), the precursors of PCs, in the secondary lymphoid organs. Consequently, a ‘wave’ of antigen-specific PBs migrates via the peripheral blood to the bone marrow and further differentiates to PCs. Such a wave perturbs homoeostasis of PCs by introducing newly generated PCs into the pool of long-lived PCs [19,23,25,26]. Only about 10% of generated PCs survive for decades [4,19,27]. This can be explained by their failure in reaching niches, whose number is limited [19,20]. Each new antigen exposure such as infection or vaccination leads to the generation of new PCs that may dislodge other PCs in competition for niches [28,22].

In this work, we aim at describing the dynamics of PCs in physiological conditions and quantifying the involved biological processes. Vaccination provides an example of an induced perturbation of PC homoeostasis and is, therefore, suited for investigating cell dynamics out of equilibrium. We develop a new mathematical model of the dynamics of healthy PCs in the bone marrow after a vaccine-induced perturbation of PC homoeostasis. The model is based on a minimum number of assumptions. It incorporates the niche as a separate cell compartment and captures known properties of PC dynamics as shown by simulations. Comparing the model with vaccination data allows quantification of the duration of PCs homing to the niche. The established mathematical framework is then applied to investigate how different rates of PC transitions into and out of the niche influence the observed PC dynamics.

According to our knowledge, it is the first mathematical model of PC dynamics based on the concept of cell competition for the niche. Several mathematical models of the long-term persistence of vaccine-specific antibodies have been proposed, assuming a simple exponential decay of the antibody levels [29–32] or a power-law decay [33–36]. Yet, these models do not distinguish among different populations of PCs [1,37]. Moreover, existing PC population models either disregard interactions between these populations [38] or do not capture the niche-related dynamics [39].

The model we propose consists of a system of four ordinary differential equations (ODEs) with a time-dependent source function to account for a wave-like inflow of vaccine-specific PCs. Replacing this time-dependent inflow by a time-discrete event results in a simplified model which can be investigated analytically. It allows explanation of the recovery of PC homoeostasis after perturbation. In particular, the model is validated regarding the fraction of vaccine-specific PCs in the niche after vaccination, and the half-lifetime of an immunity characteristic. Furthermore, we compare the model accounting for a time-dependent inflow of vaccine-specific PCs with published data from a vaccination experiment. The latter allows us to quantify the niche-related dynamics.

## 2. Mathematical modelling

The mathematical model developed in this study considers interactions of two populations of healthy PCs in the bone marrow, the vaccine-specific and vaccine-non-specific PCs. The PCs outside the niche and those inside the niche are taken into account separately. The model is based on a system of ODEs describing changes in the number of PCs per unit of time. Model variables, parameters and functions along with the basic model assumptions are listed in table 1.

**Table 1.** Description of variables, parameters and functions of the vaccination model (M) together with the model assumptions on plasma cell (PC) dynamics in the bone marrow.

symbol	description
	model variables
*x*_0_(*t*)	number of vaccine-non-specific PCs outside the niche at time *t*
*y*_0_(*t*)	number of vaccine-non-specific PCs inside the niche at time *t*
*x*_*v*_(*t*)	number of vaccine-specific PCs outside the niche at time *t*
*y*_*v*_(*t*)	number of vaccine-specific PCs inside the niche at time *t*
	model parameters
*b* > 0	transition rate of PCs into the niche
*c* > 0	transition rate of PCs out of the niche
*d* > 0	death rate of PCs outside the niche
*f* > 0	number of vaccine-non-specific PCs entering the bone marrow via the blood per unit of time
*n* > 0	difference between the numbers of PCs inside and outside the niche at homoeostasis
	model functions
*g*(*t*) ≥ 0	number of vaccine-specific PCs entering the bone marrow via the blood at time *t*
*z*(*t*)	surplus of PCs relative to homoeostasis at time *t*
	model assumptions on plasma cell dynamics in the bone marrow
*inflow*	flow of vaccine-non-specific PCs into the bone marrow is constant (*f*), and inflow of vaccine-specific PCs is time-dependent (*g*(*t*))
*death*	PCs outside the niche die at a constant rate (*d*), and PCs inside the niche do not die.
*transition*	at homoeostasis (*z*(*t*)=0) there are *n* more PCs inside the niche than outside the niche and no transition occurs. If there are more PCs outside than inside the niche relative to homoeostasis (*z*(*t*)>0), then PCs enter the niche at a constant rate (*b*). If there are more PCs inside than outside the niche relative to homoeostasis (*z*(*t*)<0) then PCs leave the niche at a constant rate (*c*)

### 2.1. Model assumptions

#### 2.1.1. Flow of cells into the bone marrow

Vaccine-independent inflow of PCs per unit of time from the peripheral blood to the bone marrow is assumed to be constant and described by the intensity *f* > 0. This is a simplification of the rather wave-like inflow [19] used for three reasons: (i) the exact shape of the wave-like inflow is not known in general. Although the number of PBs peaks at day 6 after immunization or infection independent of the antigen, the duration and magnitude of the response further depends on the persistence of the antigen [40]; (ii) there is a large variability in the number of PBs (and therefore also in the number of PCs entering the bone marrow) among individuals [40]; and (iii) although the median number of such waves can be estimated to be 30 per year [19], large deviations arise depending on the environmental ecosystem and the prevalence of infectious diseases an individual is exposed to. This is exemplified by seasonal infections such as influenza.

Besides spontaneous antigen encounters, primary immunization using vaccination induces the generation of vaccine-specific PCs which enter the bone marrow [19]. This affects homoeostasis of the resident vaccine-non-specific PCs, eventually resulting in adding the vaccine-specific PCs to the homoeostatic PC repertoire. Inflow of the vaccine-specific PCs into the bone marrow is vaccine-dependent and limited to a short time span [19,23,25,40]. Thus, it can be modelled using a time-dependent function g:[0,∞)→[0,∞), t↦g(t), which is non-zero only on a short initial time interval.

#### 2.1.2. Cell death

As the lifespan of PCs inside the niche is tens of years, which is significantly longer than the several weeks lifespan of PCs outside the niche [2,9,10,19,23,25], we assume that the PCs outside the niche die at a constant rate *d*>0. By contrast, PCs inside the niche do not die.

#### 2.1.3. Plasma cell homoeostasis and cell transitions

The total number of healthy PCs remains fairly constant in adulthood as demonstrated by constant Ig levels [7]. Thus, at homoeostasis, a constant number of the PCs inside and outside the niche is assumed. As most of the PCs are in contact with niche-associated cells [41], there are more PCs inside than outside the niche.

**Figure 1.**
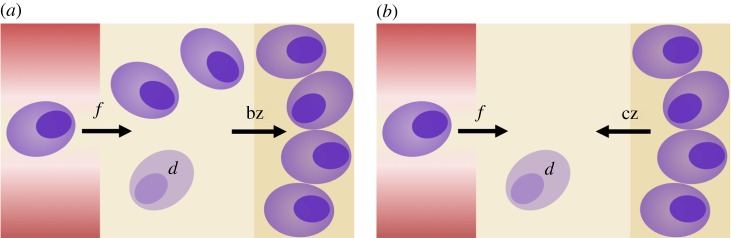
Illustration of the dynamics involved in plasma cell (PC) homoeostasis. PCs (purple) enter the bone marrow (yellow) through the peripheral blood (red) at a constant number *f*>0 per unit of time. PCs in the bone marrow are either located outside (light yellow) or inside the niche (dark yellow). PCs outside the niche die at a constant rate *d*>0 (light purple). (*a*) If there is a surplus of PCs outside the niche, i.e. *z*>0, then these PCs enter the niche at rate *b*>0. (*b*) If there is a surplus of PCs inside the niche, i.e. *z*<0, then these PCs leave the niche at rate *c*>0.

It is assumed that transitions into and out of the niche depend on the number of PCs within the bone marrow modelled by the function

z(t):=no. of PCs outside the niche−no. of PCs inside the niche+n,

representing the (positive or negative) surplus of PCs relative to homoeostasis. The parameter *n*>0 denotes the difference between the number of PCs inside and outside the niche at homoeostasis: If *z*=0, no transition occurs, and there are *n* more PCs inside than outside the niche. If the system is not at homoeostasis (e.g. owing to a vaccine-induced perturbation), cell transitions are assumed to take place. Figure 1 visualizes cell transitions considering only one PC population. If there are more PCs outside than inside the niche relative to homoeostasis (i.e. *z*(*t*)>0), then these PCs enter the niche at a constant rate *b*>0 (figure 1*a*). This involves a simultaneous enlargement of the niche [42,20], restricted by the volume of the bone marrow [4,9,43]. By contrast, if there are more PCs inside than outside the niche relative to homoeostasis (i.e. *z*(*t*)<0), then these PCs leave the niche at a constant rate *c*>0 (figure 1*b*). As a consequence, the niche is contracted. For simplification, transition rates are constant.

### 2.2. Vaccination model

Variables *x*_0_(*t*) and *y*_0_(*t*) (respectively, *x*_*v*_(*t*) and *y*_*v*_(*t*)) denote the number of vaccine-non-specific (respectively, vaccine-specific) PCs in the bone marrow outside and inside the niche at time *t*. Based on the previous assumptions, the surplus of PCs relative to homoeostasis is given by

$$z(t)=x_{0}(t)+x_{v}(t)-(y_{0}(t)+y_{v}(t))+n,\;n>0.$$

To model cell transitions, we assume that the portion of each PC type (vaccine-non-specific or vaccine-specific) within the surplus is the same as the portion of each PC type within the respective compartment where the surplus is present. This results in the transition rate function

$$\beta_{j}(z(t)):=\left\{ \begin{array}{ll} b\frac{x_{j}(t)}{x_{0}(t)+x_{v}(t)} & \text{if}\;z(t)\geq 0\\ c\frac{y_{j}(t)}{y_{0}(t)+y_{v}(t)} & \text{if}\;z(t)<0, \end{array}\;\right.$$

where *j*∈{0,*v*}. Consequently, the change in the numbers of vaccine-non-specific and vaccine-specific PCs in the bone marrow outside and inside the niche per unit of time can be described by the following system of ODEs for times *t* ≥ 0, referred to as the vaccination model:

M $$\;\begin{array}{rl} x_{0}^{\prime }(t) & \;=f-\beta_{0}(z(t))z(t)-dx_{0}(t)\\ x_{v}^{\prime }(t) & \;=g(t)-\beta_{v}(z(t))z(t)-dx_{v}(t)\\ y_{0}^{\prime }(t) & \;=\beta_{0}(z(t))z(t)\\ \text{and}\;\;y_{v}^{\prime }(t) & \;=\beta_{v}(z(t))z(t). \end{array}\}$$

Initial conditions are chosen to be non-negative, i.e. $x_{0}(0)=x_{0}^{0}\geq 0$, $x_{v}(0)=x_{v}^{0}\geq 0$, $y_{0}(0)=y_{0}^{0}\geq 0$ and $y_{v}(0)=y_{v}^{0}\geq 0$.

#### 2.2.1. Simplified vaccination model

**Figure 2.**
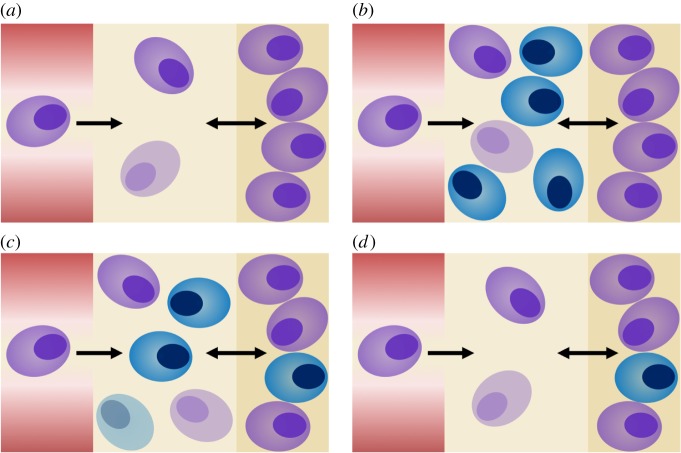
Perturbation of plasma cell (PC) homoeostasis owing to vaccine-specific PCs entering the bone marrow. (*a*) Homoeostatic distribution of vaccine-non-specific PCs (purple) in the bone marrow prior to primary vaccination. (*b*) Vaccination induces the production of a population of vaccine-specific PCs (blue). They enter the bone marrow, here depicted as blue cells appearing outside the niche. (*c*) Perturbation of PC homoeostasis causes dynamical interactions between vaccine-non-specific and vaccine-specific PCs, along with the displacement of PCs out of the niche. (*d*) The dynamics eventually lead to the re-establishment of PC homoeostasis in the bone marrow. PC homoeostasis is newly composed in that vaccine-specific PCs are added to the repertoire of PCs inside the niche.

First, we investigate the model analytically. For this purpose, we reduce the model to a system of autonomous ODEs. This can be achieved by replacing the time-dependent inflow of vaccine-specific PCs into the bone marrow, *g*(*t*), by a time-discrete event, e.g. $g(t)=x_{v}^{0}$ for *t*=0 and *g*(*t*)=0 for *t*>0. The latter can be incorporated in the model by changing the initial condition. This setting is denoted as the simplified vaccination model (see the electronic supplementary material, S2). A rigorous analysis of the simplified vaccination model is provided in the electronic supplementary material, S3. It allows us to infer the stability of PC homoeostasis and dynamic characteristics of PCs after a perturbation. As *g*(*t*) is non-zero only on a short initial time interval, the vaccination model and the simplified vaccination model coincide after an initial layer. Figure 2 provides a visulization of the PC dynamics after vaccination.

#### 2.2.2. Manifold of equilibria

In particular, the simplified model allows calculation of equilibrium states of the vaccination model. A straightforward calculation shows that these are characterized by a one-dimensional manifold of non-isolated equilibria:

$$E:=\left\{E=\left(\begin{array}{c} x_{0}^{E}\\ x_{v}^{E}\\ y_{0}^{E}\\ y_{v}^{E} \end{array}\right):E=\left(\begin{array}{c} \frac{f}{d}\\ 0\\ \frac{f}{d}+n\\ 0 \end{array}\right)+y_{v}^{E}\left(\begin{array}{c} 0\\ 0\\ -1\\ 1 \end{array}\right),0\leq y_{v}^{E}\leq \frac{f}{d}+n\right\}.$$

We note that, at an equilibrium, the total number of PCs outside and inside the niche is given by $x^{E}:=x_{0}^{E}+x_{v}^{E}=f/d$ and $y^{E}:=y_{0}^{E}+y_{v}^{E}=f/d+n$, respectively, which means that equilibria of the vaccination model correspond to PC homoeostasis (being characterized by *n* more PCs inside than outside the niche). The latter observation can be used for model parametrization as described below. Observe that the component $y_{v}^{E}$ reflects the long-term persistent number of vaccine-specific PCs inside the niche, providing a new immunity characteristic within the immune memory.

### 2.3. Model parametrization

In this section, we parametrize the models. We assume that the average total number of PCs in the human bone marrow at homoeostasis comprises 10^10^ cells (see the electronic supplementary material, S1). If not stated differently, this value is used for simulating solutions of the simplified vaccination model. To account for inter-individual variability of the PC number, the variable *ν*>0 is introduced into the model framework choosing *ν*×10^10^ cells for the total number of PCs at homoeostasis. Here, *ν* reflects the individual variation in the number of the PCs at homoeostasis relative to the average total number of PCs at homoeostasis given if *ν*=1. This parameter is important for comparing the vaccination model (M) to data from an individual patient as described below.

As the models distinguish PCs outside from those inside the niche, an assumption on the fraction of PCs outside the niche at homoeostasis is required. About 30 waves consisting of 10^7^ PCs arrive at the bone marrow per year [19]. Assuming that these waves are equally distributed over one year, such a wave arrives at the bone marrow every 12 days. A half-lifetime of PCs outside the niche of about 40 days [19,25,37] implies that, on average, the number of the PCs outside the niche is in the magnitude of at least 10^7^ cells. The latter represents 1% of the estimated total number of PCs reported in [19]. This is in line with at least 90% of all PCs being located inside the niche [41,44]. Thus, at homoeostasis, it is assumed that 99% of the total number of healthy PCs in the bone marrow are located inside the niche.

The transition rates *b* and *c* are assumed to be equal, meaning that there is no competitive advantage or disadvantage between PCs outside and inside the niche, e.g. owing to adhesion to the niche. As the migratory phase of PBs lasts for one week [19], it can be deduced that, once they have arrived at the bone marrow, the PCs enter the niche within a fraction of one week. Because the exact rates are not known, we assume *b*=*c*=1 day^−1^. This choice is supported by a sensitivity analysis as described in §§2.4 and 3.2.

Consequently, considering the models at homoeostasis prior to vaccination implies the following parametrization:

$$\begin{array}{rl} x^{E} & \;=\nu \times 10^{8}\;\mathrm{PCs},\;f=\nu \times \frac{\ln(2)}{40}\times 10^{8}\;\mathrm{PCs}\times {\mathrm{day}}^{-1},\\ y^{E} & \;=99\times \nu \times 10^{8}\;\mathrm{PCs},\;d=\frac{\ln(2)}{40}\;{\mathrm{day}}^{-1},\\ n & \;=98\times \nu \times 10^{8}\;\mathrm{PCs},\;b=c=1\;{\mathrm{day}}^{-1}. \end{array}$$

### 2.4. Parameter estimation

#### 2.4.1. Data

In this section, we compare the vaccination model (M) with data from an individual patient. A published dataset by Bernasconi *et al.* [25] is used to characterize the dynamics of vaccine-specific and vaccine-non-specific PCs after vaccination with tetanus toxoid (TT) for a follow-up of 145 days. Data for one human donor comprise two sets of immunoglobulin type G (IgG) measurements, i.e. (D1) the number of antibody-secreting cells (ASCs) secreting IgG specific to TT in the peripheral blood existing until day 23 after vaccination (see the electronic supplementary material, S1, table S1), and (D2) the dynamics of serum IgG specific to TT (see the electronic supplementary material, S1, table S2). Dataset (D1) is used as time series which describes TT-specific PBs created after a TT boost (see the electronic supplementary material, S1). We assume that the dynamics of TT-specific PBs impact proportionally on the dynamics of the flow of TT-specific PCs into the bone marrow. Then a piecewise-exponential function $\tilde{g}(t)$ which peaks at day 6 is used to characterize *g*(*t*) within the vaccination model (M), i.e.

$$g(t)=\alpha \times \tilde{g}(t)\;\text{with}\;\tilde{g}(t)=\left\{ \begin{array}{ll} a_{1}e^{b_{1}t} & \text{if}\;t\leq 6\\ a_{2}e^{b_{2}(t-6)} & \text{if}\;6\leq t\leq 23\\ 0 & \text{if}\;t>23. \end{array}\;\right.$$

The parameters *a*_1_,*a*_2_ and *b*_1_,*b*_2_ are determined on basis of dataset (D1) (see the electronic supplementary material, S4, table S3). The cut-off after day 23 is consistent with the inflow of TT-specific PCs being non-zero only on a short initial time interval. The proportionality factor *α*>0 is added to the set of parameters of the vaccination model.

Further, assuming that the production of Ig in other sites except the bone marrow is unaffected by accumulation of the PCs in the bone marrow and most of Ig is produced by PCs [16], the Ig level can be taken as a surrogate for the number of the PCs in the bone marrow. By applying a transformation of the Ig data in order to match them with the observables of the vaccination model, dataset (D2) is used as a time series for the number of TT-specific PCs (see the electronic supplementary material, S1).

#### 2.4.2. Parameter estimation

Fitting solutions of the parametrized vaccination model (M) to given data involves estimation of undetermined parameters describing the proportionality constant of the inflow of the vaccine-specific PCs (*α*) and the patient-specific number of healthy PCs at homoeostasis (expressed by *ν*). Estimation is performed using a least squares formulation to minimize the errors between the data and the model output [45], i.e.

$$\min_{\alpha ,\nu }\sum_{D}(x_{v}(t_{j})+y_{v}{\left(t_{j})-d_{j}\right)}^{2},$$

where *x*_*v*_(*t*)=*x*_*v*_(*t*;*α*,*ν*) and *y*_*v*_(*t*)=*y*_*v*_(*t*;*α*,*ν*) are solutions of the vaccination model (M) depending on the unknown parameters *α* and *ν*, and $D=\{(t_{1},d_{1}),\ldots ,(t_{n},d_{n})\},\;n\in N,$ is the set of transformed measurements of dataset (D2) with datum *j* given by *d*_*j*_ at time *t*_*j*_. Optimization is performed using the direct simplex method of Nelder & Mead [46]. Confidence intervals to a confidence level of 95% are calculated based on a linear approximation of the nonlinear model. The coefficient of determination *R*^2^ is used as the goodness-of-fit measure [45]. Computations are implemented in Mathematica (version 9, Wolfram Research).

#### 2.4.3. Sensitivity analysis

Sensitivity analysis is performed to address the influence of changes in the values for the transition rates *b* and *c* on a best fit solution [47], because there is uncertainty in their values owing to a lack of quantitative data available in the literature. For a quantification of the extent of sensitivity, the maximal relative deviation of the perturbed solution *X*^*p*^(*t*) from the best fit solution X*(*t*) is evaluated by

$${\text{max}}_{t}\frac{|X^{p}(t)-X^{*}(t)|}{X^{*}(t)}.$$

Considering this expression only within the time range of the available data (i.e. performing maximization with a constraint) allows drawing conclusions on whether different values for the investigated parameters are compatible with the obtained fit on the basis of given data.

## 3. Results

Results in §3.1 are based on mathematical analyses and simulations of the simplified vaccination model (see the electronic supplementary material, S2 and S3). For the latter, the introduced parametrization is used if not stated differently. In §3.2, the results of the parameter estimation based on the vaccination model (M) are presented.

### 3.1. Qualitative analysis and simulations of plasma cell dynamics after vaccination

#### 3.1.1. Recovery of homoeostasis

**Figure 3.**
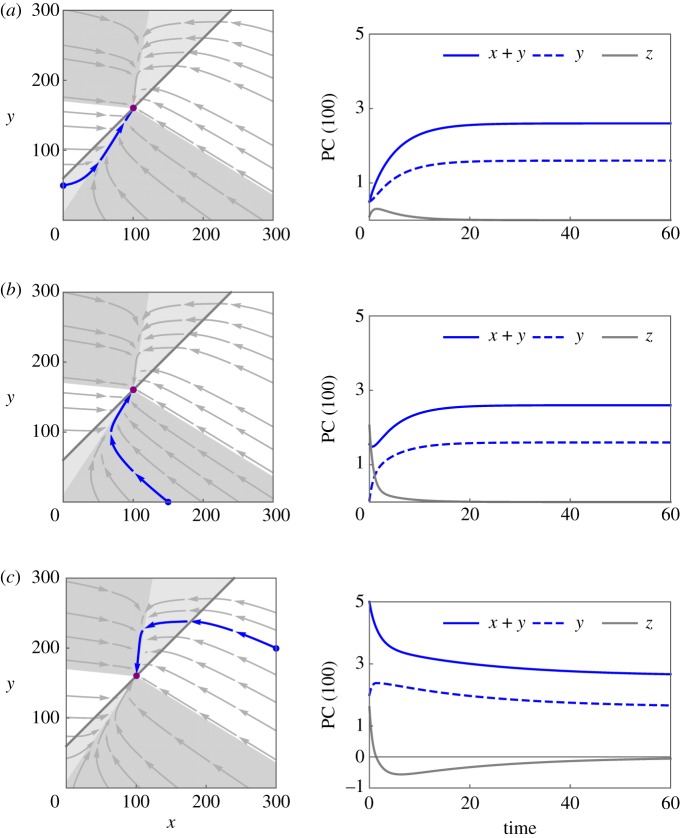
Qualitative dynamics of total plasma cell (PC) numbers outside and inside the niche. The dynamics of total numbers of PCs outside (*x*=*x*_0_+*x*_*v*_) and inside (*y*=*y*_0_+*y*_*v*_) the niche are shown. Starting in or entering either of the grey sets or dark grey sets implies that trajectories (blue) will stay therein for all times, eventually reaching homoeostasis (purple). For details, we refer to Theorem S2. Once inside of such an invariant set, trajectories never cross the line *z*=0 (dark grey), as it can be recognized by the arrows visualizing the vector field. Values of parameters are chosen as *f*=50, *n*=60, *b*=0.5, *c*=0.05, *d*=0.5 to guarantee a clear visualization of the simulations. (*a*) The trajectory starting at (0,50) implies that *z*≥0 for all times. In particular, *z* is not monotone. (*b*) The trajectory starting at (150,0) implies that *z*(*t*)≥0 for all times, and *z* is strictly monotonically decreasing. (*c*) The trajectory starting at (300,200) implies that *z* changes its sign once, i.e. *z* has exactly one root. In particular, *z* is not monotone.

Owing to the asymptotic stability of PC homoeostasis (see the electronic supplementary material, Theorem S1), injecting healthy PCs into the bone marrow and hence perturbing homoeostasis, e.g. as an effect of vaccination, leads to PC homoeostasis again. The surplus of PCs (outside or inside the niche) eventually declines to zero. To study the mode of decay, we perform a phase plane analysis (see the electronic supplementary material, Theorem S2). Figure 3 depicts the total number of PCs outside and inside the niche for three different initial settings representing perturbations of PC homoeostasis in the bone marrow associated with a surplus of PCs outside the niche. All three scenarios lead to a recovery of homoeostasis. Yet their dynamics are different depending on the initial number of PCs inside and outside the niche. Figure 3*a* depicts a scenario where PCs only enter the niche. There exists a positive time point at which the surplus of PCs outside the niche peaks, and subsequently declines to zero. In figure 3*b*, the resulting surplus is monotonically decreasing. By contrast, figure 3*c* depicts an initial number of PCs, which results in a flow of PCs into the niche owing to a high number of PCs outside the niche, and a concomitant surplus of PCs inside the niche leading to PCs being expelled from the niche and approaching homoeostasis. Such dynamics could be a consequence of a vaccination, where vaccine-specific PCs settle down in the niche and a number of resident vaccine-non-specific PCs are squeezed out of the niche. Importantly, the function *z* changes sign once. Characterizing the switching time is of particular interest as it determines the time span of vaccine-specific PCs entering the niche and the onset of the time interval during which the PCs become outcompeted. It only depends on the rate of transition into the niche and the death rate (see the electronic supplementary material, Theorem S3).

#### 3.1.2. Fraction of vaccine-specific plasma cells in the niche after vaccination

**Figure 4.**
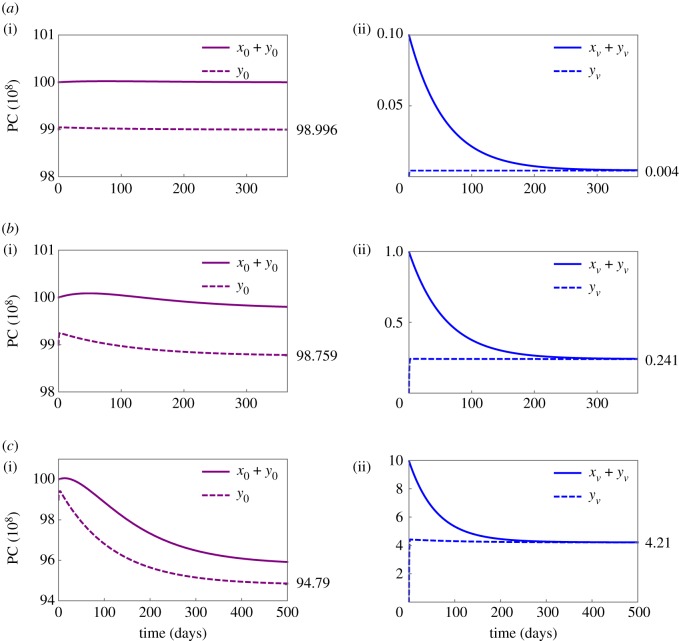
Dynamics of vaccine-specific and vaccine-non-specific plasma cells (PCs) after vaccination. Simulations of plasma cell (PC) dynamics described by the parametrized simplified vaccination model (see §2.3 and electronic supplementary material, S2). At time point zero, (*a*) 10^7^, (*b*) 10^8^ or (*c*) 10^9^ vaccine-specific PCs enter the bone marrow and perturb PC homoeostasis. (i) Dynamics of the number of vaccine-non-specific PCs in total (purple) and inside the niche (dashed purple). (ii) Dynamics of the number of vaccine-specific PCs in total (blue) and inside the niche (dashed blue). Solutions asymptotically approach an equilibrium state, reflecting that homoeostasis is recovered, and (*a*) 0.004%, (*b*) 0.243% or (*c*) 4.253% of vaccine-non-specific PCs inside the niche are replaced by vaccine-specific PCs.

Figure 4 depicts the dynamics of vaccine-non-specific and vaccine-specific PCs after a perturbation of homoeostasis consisting of 10^10^ vaccine-non-specific PCs. A perturbation is induced by 10^7^, 10^8^ or 10^9^ vaccine-specific PCs entering the bone marrow. The fraction of vaccine-specific PCs at the re-established homoeostasis is 0.004%, 0.241% and 4.21%, respectively.

To investigate how many vaccine-specific PCs need to enter the bone marrow such that 10% of those persist in the niche [19,27], homoeostasis is perturbed by a population of vaccine-specific PCs of different sizes, and the fractions of the previously generated vaccine-specific PCs in the re-established homoeostasis is calculated (table 2). The model predicts that an initial number of 3×10^7^ vaccine-specific PCs results in 33/100×10^7^ persisting PCs, i.e. about 10% of the generated vaccine-specific PCs. It is worth noting that this result is based on the average total number of vaccine-non-specific PCs at homoeostasis (i.e. 10^10^). Assuming a different cell count because of inter-individual heterogeneity in the number of PCs at homoeostasis would result in different numbers of vaccine-specific PCs necessary to reproduce the above-mentioned observation.

**Table 2.** Portion of persisting vaccine-specific plasma cells (PCs) after a vaccine-induced perturbation of the homoeostasis. (Using the parametrized simplified vaccination model, homoeostasis consisting of 10^10^ vaccine-non-specific PCs is perturbed by a population of vaccine-specific PCs entering the bone marrow. This results in a re-established homoeostatic arrangement of PCs, which includes only a fraction of the previously generated vaccine-specific PCs.)

number of	persisting
vaccine-specific PCs	vaccine-specific PCs
10^4^	<0.05%
10^5^	0.05%
10^6^	0.48%
10^7^	4.39%
2×10^7^	8.04%
3×10^7^	11.13%
4×10^7^	13.78%
5×10^7^	16.08%
6×10^7^	18.08%
7×10^7^	19.85%
8×10^7^	21.42%
9×10^7^	22.82%
10^8^	24.08%
10^9^	42.10%

#### 3.1.3. Half-lifetime of an immunity characteristic

To investigate the number of vaccine-non-specific antigen encounters needed to reduce the newly established immunity characteristic to 50%, we assume that vaccination introduced 33/100×10^7^ vaccine-specific PCs within a pool of 10^10^ PCs, and interpret vaccine-non-specific antigen encounters as vaccinations with antigen different from that induced by the previous vaccines (see the electronic supplementary material, S2). As a result, about 480 vaccinations are necessary for the desired reduction. Assuming that a maximum of 30 waves of PCs arrive at the bone marrow owing to natural antigenic adaptions to humoral immunity, this translates into (at least) 16 years, which is in line with Radbruch *et al.* [19] in terms of the order of magnitude.

### 3.2. Comparison of plasma cell dynamics to vaccination data

#### 3.2.1. Duration of plasma cells homing to the niche

**Figure 5.**
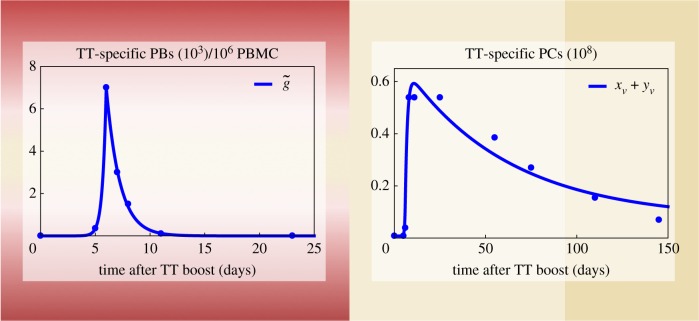
Comparison of the vaccination model (M) to data. Data consist of numbers of plasmablasts (PBs) per 10^6^ peripheral blood mononuclear cells (PBMCs) and plasma cells (PCs) specific to tetanus toxoid (TT) after TT boost. (*a*) Inflow of TT-specific PCs is assumed to be proportional to the number of PBs present in the peripheral blood. A piecewise-exponential function with peak at day 6 ($\tilde{g}$) is fitted to measurements (see the electronic supplementary material, S4, table S3). (*b*) Resulting best fit of the transformed measurements describing the total number of TT-specific PCs by means of the parametrized vaccination model (M) (see the electronic supplementary material, S4, table S4).

Figure 5 depicts solutions of the vaccination model (M), which fit the vaccination data best (*R*^2^=0.978; see the electronic supplementary material, S4, tables S3 and S4). Whereas the number of PBs in the peripheral blood peaks at day 6 after the boost as assumed by the data, the model indicates that the number of TT-specific PCs in the bone marrow peaks at day 10 after the boost. It should be noted that data suggest a plateau in the response of TT-specific PCs between day 8 and day 25 rather than a peak. This phenomenon is not captured by our model and might point towards its limitation. However, such a plateau is not seen to be consistent with PCs entering the niche in a wave-like inflow [19]. It might be an artefact, e.g. caused by an insufficient resolution of the data lacking relevant time points within the corresponding time span.

**Figure 6.**
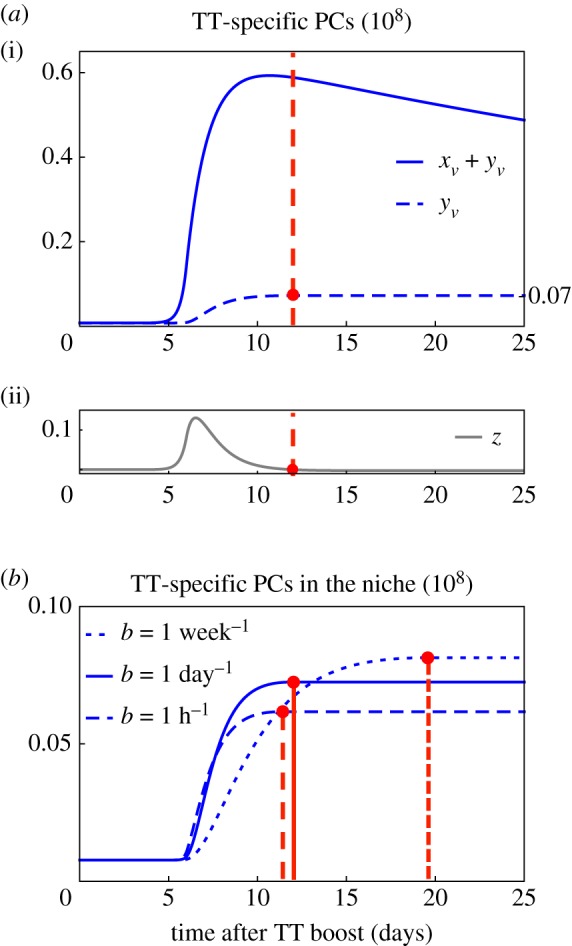
Analysing the dynamics of tetanus-toxoid (TT)-specific plasma cells (PCs). The fit for the total number of TT-specific PCs is analysed with respect to the dynamics of TT-specific PCs entering or leaving the niche. (*a*)(i) Fit for the total number of TT-specific PCs (bold line) and corresponding solutions for the number of TT-specific PCs inside the niche (dashed line). (ii) Dynamics of the surplus of PCs relative to homoeostasis. At day 12 after the TT boost, the function *z* changes sign from positive to negative (red point), meaning that settlement of TT-specific PCs inside the niche takes place until day 12. After day 12, few TT-specific PCs leave the niche. This is likewise visualized in (i) by an almost constant number of 0.07×10^8^ TT-specific PCs inside the niche after day 12. (*b*) The obtained fit for the total number of TT-specific PCs inside the niche is analysed with respect to variations in the transition rate of PCs into the niche. *b*=1 day^−1^ (bold line) is altered by increasing it to 1 h^−1^ (dashed line), and by decreasing it to 1 week^−1^ (dotted line). Maximal relative deviations from the fit amount for 15%. Red points mark times at which the function *z* changes sign from positive to negative. Thus, a faster transition of PCs into the niche implies a faster termination of PCs homing to the niche, and a lower number of TT-specific PCs residing inside the niche.

The 4 day interval between the peaks indicates that a wave of TT-specific PCs needs 4 days to pass from the peripheral blood to the bone marrow. In accordance with the qualitative results, the vaccine-induced perturbation of homoeostasis causes a surplus of TT-specific and TT-non-specific PCs in the bone marrow outside the niche, and consequently, a transition of PCs into the niche compartment. At the switching time, here at day 12 after the boost, transition reverses and PCs leave the niche, eventually approaching homoeostasis consisting of about 7×10^6^ cells. The number of TT-specific PCs is fairly unchanged after the switching time (figure 6*a*). This can be explained by a small fraction of TT-specific PCs inside the niche compared to TT-non-specific PCs. Thus, competition starting about two weeks after the TT boost mainly affects TT-non-specific PCs (see also the electronic supplementary material, S4, figure S1).

#### 3.2.2. Effect of altered transition rates

Previous results have been obtained under the assumption that PCs enter and leave the niche within one day (i.e. *b*=1 per day and *c*=1 per day, respectively). Varying the transition rates within a biologically plausible range, i.e. assuming faster transitions into or out of the niche (*b*=1 per hour or *c*=1 per hour), or slower transitions into or out of the niche (*b*=1 per week or *c*=1 per week) results in negligible changes in the total number of TT-specific PCs: the maximal relative deviation from the fit is about 8% for variations in *b*, and 0.003% for variations in *c* (see the electronic supplementary material, S4, figure S2), both considered within the time range of the data. Thus, the transition rates could not have been quantified on the basis of the data, because different values only slightly affect the observed model output, in turn justifying fixing the *b* and *c* values. Indeed, additional estimation of *b* increases parameter uncertainty (see the electronic supplementary material, S4, table S5). By contrast, changes in the transition rate *b* affect the dynamics of the not observed count of TT-specific PCs inside the niche to a larger degree, i.e. with a maximal relative deviation from the fit of about 15% (figure 6*b*). Different values of *b* are in turn related to different levels of TT-specific PCs at the re-established homoeostasis. Durations for TT-specific PCs homing to the niche are different in that homing takes about 11 days assuming a fast transition of PCs entering the niche of one hour, and about 20 days for a slow transition of one week. For the considered parameter values, a faster transition of PCs into the niche implies a faster termination of PCs homing to the niche, and a lower number of TT-specific PCs residing inside the niche.

It is of note that for even faster transitions of PCs into the niche (i.e. *b*=1 per minute), the results do not significantly differ compared to the previous case (i.e. *b*=1 per hour). Consequently, such faster transition would not be associated with any additional value for the population of TT-specific PCs, e.g. obtaining a higher level of cells at the re-established PC homoeostasis. On the other hand, a further slowdown in the transition of PCs into the niche (i.e. *b*=1 per month) results in a duration of about 40 days for PCs homing to the niche, and in a lower level of TT-specific PCs at the re-established homoeostasis compared to *b*=1 per day.

## 4. Discussion

We introduced, to our knowledge, the first mathematical model describing PC dynamics, which accounts for niche-dependent cell dynamics. Mathematical analysis implies that homoeostasis is eventually re-established after perturbations owing to vaccination, in agreement with fairly stable levels of Ig during a healthy individual’s lifespan [7,19,22,28]. Owing to a wave of vaccine-specific PCs arriving at the bone marrow [19], the number of PCs outside the niche increases. As a consequence, PCs are pushed into the niche, which becomes enlarged [4,9,43]. The duration of PCs entering the niche is seen independent of the number of vaccine-specific PCs entering the bone marrow. Simulations indicate that not only vaccine-specific but also vaccine-non-specific PCs enter the niche, as observed by Bernasconi *et al.* [25]. This could be owing to the burst of vaccine-specific PCs shepherding vaccine-non-specific PCs into the bone marrow; this process potentially is a consequence of simultaneous activation of antibody-secreting cells by further antigens. The subsequent reduction in the number of PCs outside the niche implies a surplus of PCs inside the niche relative to homoeostasis, in turn resulting in a contraction of the niche compartment. Such contraction was experimentally observed following Salmonella infection in mice [48]. As a consequence, PCs are expelled from the niche. The magnitude of the competition-caused loss of a particular PC population is proportional to its relative frequency in the total PC population inside the niche. As the number of vaccine-specific PCs arriving at the bone marrow is two orders of magnitude smaller than the total number of PCs in the bone marrow [19], competition does not affect the vaccine-specific PCs. This is in line with vaccine-non-specific PCs found in the peripheral blood after immunization [19,21,26,49]. Dislocated PCs die because they are no longer being supported by the niche, and this leads to a reduction in the antibody levels [19]. By contrast, if the model assumed that the PCs were constantly leaving the niche, this would yield decreasing antigen-specific PC populations, and stable immune characteristics would never become manifest.

Using the parametrized simplified model, we have shown that reported observations can be reproduced, including the reduction of an immunity characteristic [19]. The simulated fraction of vaccine-specific PCs at homoeostasis is in line with biological observations reporting that 0.1–1% of the pre-existing PC population is replaced by PCs created owing to an antigen encounter [19]. Variations in antibody immune response patterns within and among individuals may result from inter-individual variability in the number of PCs at homoeostasis as well as in the number of antigen encounters per year, each generating different numbers of PCs entering the bone marrow depending on the strength of the initial stimulation and the presence of ongoing stimuli [1,37]. The vaccination model can take into account both sources of individual variability by choosing an individual value for the parameter *ν*, and by defining an individual function *g*(*t*) for the time-dependent inflow of vaccine-specific PCs, respectively.

Comparing the solutions of the vaccination model to a patient dataset demonstrates that the proposed framework is first compatible with the dynamics of TT-specific PCs observed within five months after vaccination. Second, comparison of the timing of the peaks of the number of TT-specific PCs in the peripheral blood and in the bone marrow results in a delay of 4 days, thus characterizing the ‘speed’ of the ‘wave’ of TT-specific PCs arriving at the bone marrow. This is consistent with the duration of the migratory phase of PBs lasting for one week [19]. Third, TT-specific PCs enter the niche at latest 12 days after the TT boost. This suggests that the time of generating long-lived PCs (i.e. PCs residing in the niche) is short (approximately two weeks) relative to the time span of the vaccination-induced dynamics (months), represented here by measurements taken until 145 days after the boost. Whereas the decline of the TT-specific PCs was previously supposed to be consistent with death of long-lived PCs [25], modelling supports the hypothesis that such turnover is owing to death of PCs *outside the niche*. Fourth, different biologically plausible magnitudes of the rate of transition into the niche (hours, days, weeks) affect the duration of homing of the TT-specific PCs to the niche (11–20 days) and their number in the niche (with a maximal relative deviation from the best fit of about 15%), but not their magnitudes.

The vaccination model is based on a number of assumptions and simplifications regarding the underlying biological processes. Most importantly, the niche is defined as a clearly separated compartment within the bone marrow, simplifying the complexity of the bone marrow micro-environment. Transitions of PCs into and out of the niche are modelled using a nonlinearity depending only on the number of PCs in the bone marrow, and rates are assumed to be constant. However, cell migration might more realistically be guided by a chemokine gradient. For example, it is known that PCs express the CXC-chemokine receptor 4 and exhibit increased sensitivity to its ligand CXC-chemokine ligand 12, which mediates entry of PCs into the bone marrow [50,51]. A more detailed description of the niche and the mechanisms involved in PC mobilization would either need a deeper understanding of the functional activities of the niche-defining cell populations and biochemical factors regulating PC migration, or profound hypotheses. In turn, this would increase the complexity of the model (including spacial dispersion of chemokine molecules) and simultaneously the need for more assumptions on the involved processes as, e.g. the signals involved in PC survival are yet unclear [19].

Taking this a step further, the model parameters might be subject to a nonlinear feedback and hence time dependent. In particular, the assumption of a constant inflow of vaccine-non-specific PCs into the bone marrow disregards the time-dependent frequencies and characteristics of antigen encounters [19,40]. These might have an impact on the dynamics of specific PCs residing inside the niche, as each antigen encounter induces the generation of new PCs which dislocate the pre-existing PCs. This implies that the number of TT-specific PCs in the niche declines rather than remains in an equilibrium state as predicted by our model. While this effect of competition would be important in the long run (i.e. after decades), in short term, it has a negligible impact on TT-specific PCs owing to their relatively small count within the total population of PCs inside the niche.

Our model can be extended to investigate more complex cell–niche interactions in health and disease. Considering the population of healthy PCs as a dynamical system, and external actions or disease as large perturbations of its homoeostatic equilibrium state, our mathematical model allows investigation of the mechanisms of physiological and pathological states of PCs. In particular, it can be expanded to describe the accumulation of malignant PCs in multiple myeloma, the second most common haematological malignancy [52,20], as a perturbation of the dynamics of healthy PCs comparable to a vaccine-induced perturbation.

## Supplementary Material

Supplement “Quantification of plasma cell dynamics using mathematical modelling”

## Data accessibility

This study is based on existing data that are available at https://doi.org/10.1126/science.1076071 [25]. Further documentation about data processing are available in the Mathematical Modelling section and as supplementary information accompanying this paper. No new data were created during this study.
