# Supplementary material for: Quantification of plasma cell dynamics using mathematical modelling
Source: R Soc Open Sci. 2018 Jan 24;5(1):170759. doi: 10.1098/rsos.170759 (PMC5792876; doi:10.1098/rsos.170759)
Supplement: Supplement “Quantification of plasma cell dynamics using mathematical modelling” [file rsos170759supp1.pdf]

Supplement

Quantification of plasma cell dynamics using mathematical modelling

Marcel Mohr[1,2], Dirk Hose[2], Anja Seckinger[2], Anna Marciniak-Czochra[1]

1 Heidelberg University, Institute of Applied Mathematics, BIOQUANT and IWR, Heidelberg, Germany

2 Heidelberg University Hospital, Medical Clinic V, Heidelberg, Germany

Contents

S1 Data and their transformation **2**

S2 Simplified vaccination model **5**

S3 Mathematical results **7**

S4 Parameter estimation **13**

S1 Data and their transformation

Table S1. Dataset (D1): Dynamics of circulating antibody-secreting cells (ASCs) secreting IgG to tetanus toxoid (TT) after TT boost. ASCs are measured per 10^6 peripheral blood mononuclear cells. Data were obtained from [1].

**Days after TT boost**	**ASCs secreting TT-IgG**
0	0
5	350
6	7000
7	3000
8	1500
11	100
23	0.2

Table S2. Dataset (D2): Dynamics of serum IgG to tetanus toxoid (TT). Serum anti-TT IgG is measured in μg per ml. Data refer to the same donor as in Table S1 and were obtained from [1].

**Days after TT boost**	**Serum anti-TT IgG**
0	1
5	1
6	5
8	70
11	70
25	70
55	50
75	35
110	20
145	9

A published dataset by Bernasconi et al. [1] is used to characterise the dynamics of vaccine-specific and vaccine-nonspecific plasma cells (PCs) after vaccination with tetanus toxoid (TT) for a follow-up of 145 days. Data for one human donor comprise two sets of immunoglobulin type G (IgG) measurements, i.e. (D1) the number of antibody-secreting cells (ASCs) secreting IgG specific to TT in the peripheral blood (see Table S1), and (D2) the dynamics of serum IgG specific to TT (see Table S2). Dataset (D1) is used as time series which describes TT-specific PBs created after TT boost, and dataset (D2) is used as time series for the number of TT-specific PCs by applying a transformation of the Ig data in order to match them with the observables of the vaccination model (M).

From estimates based on large cohorts of adult normal-weighted healthy individuals it can be deduced that about 95% of the Ig concentrations per litre of blood lie within the interval $(7.65, 20.13)$ (in g/l) [2]. The average Ig concentration can be estimated to comprise approximately 13 g/l with fractions of common Ig types given by 10 g/l IgG, 2 g/l IgA and 1 g/l IgM [2, 3]. Ig types with marginal incidence (IgD, IgE) are neglected. The fraction of an Ig type corresponds to the fraction of PCs secreting that particular Ig type [4]. Estimates for the average total number of PCs in the bone marrow are deduced from two different approaches:

1. **Proposed estimates:** The total number of PCs in the human bone marrow is estimated to be about 10^9 cells [5]. The bone marrow cellularity is estimated to comprise about 10^{12} cells [6]. Assuming a fraction of PCs of $0.25 - 1\%$ [5, 7], the total number of PCs can be calculated to be in the range of $2.5 \cdot 10^9 - 10^{10}$ cells. A comparable result using that the number of PCs in the bone marrow comprises about $7.5 \cdot 10^9$ cells per litre of bone marrow can be obtained [8]. With a total bone marrow volume of $1.6 - 4.0$ litres [9], this implies an estimate of PC counts in the range of $1.2 - 3 \cdot 10^{10}$ cells. Summarising reported values yields that the average total number of PCs in the human bone marrow lies within $2.5 \cdot 10^9 - 3 \cdot 10^{10}$ cells.

2. **Estimates based on Ig synthesis rates of PCs:** The total amount of IgG at homoeostasis is assumed to be 50 g. The half-life time of IgG molecules can be estimated to be 20 days [10–12]. IgG synthesis rate is in a range of 20 pg - 35 pg per PC per day with mean of 26 pg per PC per day [13]. In order to relate the synthesis rates to the total number of PCs, we define $G(t)$ as the amount of IgG molecules (in gramme) at time t (in days). The production and degradation of IgG is modelled as $G'(t) = sC(t) - d_g G(t)$, where $C(t)$ is the total number of the IgG-producing PCs at time t, s is the synthesis

rate, and d_g is the degradation rate of IgG molecules (which is given by the reciprocal of the half-life time multiplied by ln(2)). At homoeostasis, it holds $sC = d_g G$, where C and G are the total amount of IgG at equilibrium (which is known), and the total number of the IgG-producing PCs at equilibrium, respectively. Using this mean synthesis rate results in

$$C = \frac{d_g G}{s} = \frac{\ln(2)}{26 \cdot 20} \cdot 50 \cdot 10^{12} \text{ PCs} \approx 6.7 \cdot 10^{10} \text{ PCs}.$$

Taking into account that C reflects 10/13 of the total number of PCs [2, 3], the total number of PCs can be estimated to be comprised of $8.7 \cdot 10^{10}$ cells.

Combining both ways of estimation results in a span of $2.5 \cdot 10^9 - 8.7 \cdot 10^{10}$ cells. Taking the mean order of magnitude motivates the assumption that the average total number of PCs in the human bone marrow comprises 10^{10} cells. Consequently, a measurement of 1 g/l Ig in the serum is equivalent to $1/13 \cdot 10^{10}$ PCs in the bone marrow.

S2 Simplified vaccination model

In this section, we consider the following system of ordinary differential equations (ODEs) for times $t \geq 0$, referred to as simplified vaccination model,

$$
\begin{aligned}
x_0'(t) &= f - \beta_0(z(t))z(t) - dx_0(t) \\
x_v'(t) &= \beta_v(z(t))z(t) - dx_v(t) \\
y_0'(t) &= \beta_0(z(t))z(t) \\
y_v'(t) &= \beta_v(z(t))z(t).
\end{aligned}
\tag{SM1}
$$

If not stated differently, without loss of generality, we consider initial conditions denoted as $x(0) = x^0 \geq 0,\ y(0) = y^0 \geq 0$ and $x_0^0(0) = x_0^0 \geq 0,\ x_v^0(0) = x_v^0,\ y_0^0(0) = y_0^0 \geq 0,\ y_v^0(0) = y_v^0 \geq 0$, respectively. Note that system (SM1) corresponds to system (M) with $g \equiv 0$.

Reduced model

Since

$$
\begin{aligned}
(x_0 + x_v)'(t) &= x_0'(t) + x_v'(t) \\
&= f - \beta_0(z(t))z(t) - dx_0(t) - \beta_v(z(t))z(t) - dx_v(t) \\
&= f - (\beta_0(z(t)) + \beta_v(z(t)))\, z(t) - d\,(x_0(t) + x_v(t))
\end{aligned}
$$

and

$$
\begin{aligned}
(y_0 + y_v)'(t) &= y_0'(t) + y_v'(t) \\
&= \beta_0(z(t))z(t) + \beta_v(z(t))z(t)
\end{aligned}
$$

the dynamics of the total numbers of PCs outside and inside the niche, $x(t) := x_0(t) + x_v(t)$ and $y(t) := y_0(t) + y_v(t)$, respectively, are described by the reduced model

$$
\begin{aligned}
x'(t) &= f - \beta(z(t))z(t) - dx(t) \\
y'(t) &= \beta(z(t))z(t)
\end{aligned}
\tag{SM2}
$$

with

$$
\beta(z(t)) := \begin{cases} b & \text{if } z(t) \geq 0 \\ c & \text{if } z(t) < 0. \end{cases}
$$

Initial conditions relate as $x^0 = x_0^0 + x_v^0$ and $y^0 = y_0^0 + y_v^0$. This allows investigating x and y in the phase plane, and characterising the niching dynamics with respect to the function z given different initial conditions.

The reduced model possesses exactly one equilibrium as it can be seen by setting the right-hand side of system (SM2) equal to zero, and solving for x and y. A straightforward calculation yields

$$E_h := \left(x^{E_h}, y^{E_h}\right) := \left(\frac{f}{d}, \frac{f}{d} + n\right)$$

denoted as healthy equilibrium. Observe that E_h corresponds to PC homoeostasis, which is characterised by n more PCs inside than outside the niche.

Vaccine-induced perturbation

Characteristics of the inflow kinetics of PCs due to vaccination (described by $g(t)$ in the vaccination model (M)) vary within and among individuals [5, 14]. Lacking data describing those kinetics imply the necessity for approximating the time-dependent inflow dynamics using a simpler formulation. A vaccine-induced perturbation of PC homoeostasis due to primary immunisation can be implemented into the simplified vaccination model (SM1) framework using a time-discrete formulation, which can be interpreted as initial condition. Assuming that the system is at equilibrium prior to vaccination, and the perturbation occurs at time T, such perturbation may be characterised as follows: For $0 \leq t < T$, it is $(x_h(t), x_v(t), y_0(t), y_v(t)) = (x^{E_h}, 0, y^{E_h}, 0)$, meaning that the system is at healthy equilibrium, again denoted as E_h. At $t = T$, it is $(x_h(t), x_v(t), y_0(t), y_v(t)) = (x^{E_h}, x_v^0, y^{E_h}, 0)$, where $x_v^0 > 0$ corresponds to the amount of vaccine-specific PCs perturbing homoeostasis.

Such formalism enables modelling vaccine boosts, i.e. further administrations of a vaccine following earlier doses. Prior to the boost, the simplified vaccination model (SM1) is at homoeostasis, which is characterised by a respective homoeostatic arrangement of vaccine-specific and vaccine-nonspecific PCs located on the manifold $\mathcal{E}$ (see main article). In particular, to investigate the number of vaccinations of antigens different from a previously used vaccine needed to reduce the newly established immunity characteristic to 50%, an iterative procedure is accomplished as follows: Starting at a healthy equilibrium $E_0 \in \mathcal{E}$ with components $x_0^{E_0}$, $x_v^{E_0}$ and $y_0^{E_0}$, $y_v^{E_0}$, the j-th boost is such that the $(j-1)$-th healthy equilibrium E_{j-1} is perturbed by a certain number N of PCs, i.e. $x_0^0 = x_0^{E_{j-1}} + N$, $x_v^0 = x_v^{E_{j-1}}$ and $y_0^0 = y_0^{E_{j-1}}$, $y_v^0 = y_v^{E_{j-1}}$. Here, vaccine-nonspecific PCs are generated by antigens different from the previously administered vaccine. This leads to a new healthy equilibrium $E_j \in \mathcal{E}$, and a further perturbation is carried forward. In accordance with the obtained results (see main article), we choose $N = 3 \cdot 10^7$ and the iterative procedure is terminated as soon as $y_v^{E_j} \leq 1/2 \cdot 33/100 \cdot 10^7$.

S3 Mathematical results

The presented models belong to the class of piecewise-smooth dynamical systems with switching manifold given by the set $z(t) = 0$ [15]. Existence and uniqueness of solutions for given initial conditions can be guaranteed due to Lipschitz continuity of the vector fields. The latter can be shown by straightforward calculations [16, 17]. Likewise, elementary calculations applying the method of invariant sets yields non-negativity of solutions for biologically plausible parameter settings [17, 18]. Analytical investigations in case of the vaccination model (M) are complicated by the non-autonomous nature of the system, and are not in the scope of this study. Instead, the simplified vaccination model (SM1) and its reduced variant (SM2) are considered.

Stability of plasma cell homoeostasis

The healthy equilibrium E_h of the reduced model and the manifold $\mathcal{E}$ of the (simplified) vaccination model are located on the switching manifold. Thus, linearised stability theory as applied for smooth dynamical systems [19] may lead to incorrect conclusions [15,20]. Instead, providing a common Lyapunov function, i.e. a function that is Lyapunov for each of the vector fields defining the systems dynamics in each of the phase space regions separated by the switching manifold guarantees global asymptotic stability of the homoeostatic equilibria [21].

Theorem S1.

(a) The healthy equilibrium E_h of the reduced model (SM2) *is globally asymptotically stable.*

(b) The manifold of non-isolated equilibria $\mathcal{E}$ of the simplified vaccination model (SM1) *is globally asymptotically stable, i.e. solutions asymptotically approach $\mathcal{E}$.*

Proof. The function $V : [0, \infty) \times [0, \infty) \to [0, \infty)$ defined by

$$V(x, y) := \frac{1}{2d}\left(x + y - 2\frac{f}{d} - n\right)^2 + \frac{1}{2\max\{b, c\}}\left(y - \frac{f}{d} - n\right)^2$$

is a strict Lyapunov function of system (SM2) for all $(x, y) \neq E_h$. The proof is left to the reader. Thus, E_h is globally asymptotically stable, which shows (a).

It holds that $x(t) = x_0(t) + x_v(t)$, $y(t) = y_0(t) + y_v(t)$, where $x(t)$ and $y(t)$ are solutions of system (SM2). Due to (a), it follows that

$$\lim_{t\to\infty} (x_0(t) + x_v(t)) = x^{E_h} = \frac{f}{d}$$

$$\lim_{t\to\infty} (y_0(t) + y_v(t)) = y^{E_h} = \frac{f}{d} + n$$

and in particular $\lim_{t\to\infty} z(t) = 0$. It remains to show that $\lim_{t\to\infty} x_v(t) = 0$. Then, every solution of system (SM1) asymptotically approaches $E = (x_0^E, x_v^E, y_0^E, y_v^E) \in \mathcal{E}$ due to

$$x_0^E + x_v^E = 0 + \frac{f}{d}$$
$$y_0^E + y_v^E = \left(\frac{f}{d} + n - y_v^E\right) + y_v^E = \frac{f}{d} + n.$$

Let $\varepsilon > 0$ arbitrary but fixed. Since $\lim_{t\to\infty} z(t) = 0$, it follows that for $0 < \tilde{\varepsilon} < \frac{\varepsilon d}{2\max\{b,c\}}$ there exists a $\tilde{T} > 0$ such that $|z(t)| < \tilde{\varepsilon}$ for all $t \geq \tilde{T}$. Thus, for all $t \geq \tilde{T}$, it is $x_v'(t) = -\beta_v(z(t))z(t) - dx_v(t) < \max\{b,c\}\tilde{\varepsilon} - dx_v(t)$. Next, consider the following ordinary differential equation with initial condition,

$$w'(t) = \max\{b,c\}\tilde{\varepsilon} - dw(t)$$
$$w(\tilde{T}) \geq x_v^0 \text{ and } w(\tilde{T}) > \frac{\max\{b,c\}\tilde{\varepsilon}}{d}$$

for all $t \geq \tilde{T}$. It follows that

$$w(t) = \underbrace{\frac{\max\{b,c\}\tilde{\varepsilon}}{d}}_{<\varepsilon/2} + e^{-d(t-\tilde{T})}\left(w(\tilde{T}) - \frac{\max\{b,c\}\tilde{\varepsilon}}{d}\right)$$

for all $t \geq \tilde{T}$. Using the comparison principle [16] implies $x_v(t) < w(t) < \frac{\varepsilon}{2} + \frac{\varepsilon}{2} = \varepsilon$. Observe that the first inequality is true for all $t \geq \tilde{T}$, whereas the second holds for all $t \geq T$, where $T > \tilde{T}$ is chosen such that the second term in $w(t)$ is smaller than $\frac{\varepsilon}{2}$. This can be achieved since the latter is positive and exponentially decreasing in time. Due to non-negativity of $x_v(t)$ for all $t \geq 0$, it follows that $\lim_{t\to\infty} x_v(t) = 0$. This proves (b). □

Biological consequences

Biologically, Theorem S1 implies that any non-malignant perturbation of PC homoeostasis eventually results in PC homoeostasis again with the same number of PCs outside and inside the niche. In particular, this holds true for a vaccination-induced perturbation, where the composition of the recovered homoeostasis depends on the number of vaccine-specific PCs arriving in the bone marrow.

Plasma cell dynamics after a perturbation of homoeostasis

Characterisation of the dynamics of $z(t)$ is provided using the concept of invariant sets [18]. The below defined sets $\mathcal{R}_1, \mathcal{R}_2$ ($\mathcal{M}_1, \mathcal{M}_2$) correspond to the gray (dark gray) sets visualised in Figure 3 (see main article).

Theorem S2.

(a) Consider the sets $\mathcal{R}_1$ *and* $\mathcal{R}_2$ *defined by*

$$\mathcal{R}_1 := \{(x,y) \in \mathbb{R}^2 \colon R_1(x,y) \le 0\} \cap \{(x,y) \in \mathbb{R}^2 \colon z \ge 0\}$$
$$\mathcal{R}_2 := \{(x,y) \in \mathbb{R}^2 \colon R_2(x,y) \le 0\} \cap \{(x,y) \in \mathbb{R}^2 \colon z \le 0\}$$

where $R_i : \mathbb{R}^2 \to \mathbb{R},\ i = 1,2,$ *are given by*

$$R_1(x,y) := y + \frac{\sqrt{4b^2+d^2}-d}{2b}x - \Big(1 + \frac{\sqrt{4b^2+d^2}-d}{2b}\Big)\frac{f}{d} - n$$
$$R_2(x,y) := -y - \frac{\sqrt{4c^2+d^2}-d}{2c}x + \Big(1 + \frac{\sqrt{4c^2+d^2}-d}{2c}\Big)\frac{f}{d} + n.$$

Then $\mathcal{R}_1$ *and* $\mathcal{R}_2$ *are positively invariant with respect to the flow of system* (SM2)*, i.e. solutions starting in* $\mathcal{R}_1$ *or* $\mathcal{R}_2$*, respectively, remain therein for all times. In particular, the function* $z(t)$ *switches its sign at most once.*

(b) Consider the sets $\mathcal{M}_1 \subset \mathcal{R}_1$ *and* $\mathcal{M}_2 \subset \mathcal{R}_2$ *defined by*

$$\mathcal{M}_1 := \{(x,y) \in \mathbb{R}^2 \colon R_1(x,y) \le 0\} \cap \{(x,y) \in \mathbb{R}^2 \colon M_1(x,y) \le 0\}$$
$$\mathcal{M}_2 := \{(x,y) \in \mathbb{R}^2 \colon R_3(x,y) \le 0\} \cap \{(x,y) \in \mathbb{R}^2 \colon M_2(x,y) \le 0\}$$

where $M_i : \mathbb{R}^2 \to \mathbb{R},\ i = 1,2,$ *are given by*

$$M_1(x,y) := y - \Big(1 + \frac{d}{2b}\Big)x - n + \frac{f}{2b}$$
$$M_2(x,y) := -y + \Big(1 + \frac{d}{2c}\Big)x + n - \frac{f}{2c}.$$

Then $\mathcal{M}_1$ *and* $\mathcal{M}_2$ *are positively invariant with respect to the flow of system* (SM2)*, i.e. solutions starting in* $\mathcal{M}_1$ *or* $\mathcal{M}_2$*, respectively, remain therein for all times. In that case, the function* $z(t)$ *is monotonically decreasing or increasing, respectively.*

Proof. First, consider the set $\mathcal{R}_1$. Let $(x,y) \in \partial\mathcal{R}_1 \setminus E_h$. The boundary of $\mathcal{R}_1$ splits up into two parts, i.e. (i) $R_1(x,y) = 0$ with $x > \frac{f}{d}$, and (ii) $z = z(x,y) = 0$ with $x < \frac{f}{d}$. Note that if $x = \frac{f}{d}$, then $y = \frac{f}{d} + n$, and thus $(x,y) = E_h$. For the first part, a straightforward calculation shows that

$$\frac{d}{dt}R_1(x,y) = b(x-y+n) + \frac{\sqrt{4b^2+d^2}-d}{2b}\Big(f - b(x-y+n) - dx\Big) \overset{(R_1=0)}{=} 0,$$

and for the second part, it is

$$\frac{d}{dt}z(x,y) = b(x-y+n) - \Big(f - b(x-y+n) - dx\Big) \overset{(z=0)}{=} dx - f < 0.$$

This implies positive invariance of the set $\mathcal{R}_1$. The proof of the assertion for $\mathcal{R}_2$ is similar and left to the reader. A change in sign of $z(t)$ is only possible for a solution starting in $\mathbb{R}^2 \setminus (\mathcal{R}_1 \cup \mathcal{R}_2)$. Let $(x^0, y^0) \in \mathbb{R}^2 \setminus (\mathcal{R}_1 \cup \mathcal{R}_2)$. Then, the corresponding trajectory $(x(t), y(t))$ eventually enters $\mathcal{R}_1 \cup \mathcal{R}_2$ as $t \to \infty$. This is due to global asymptotic stability of $E_h \in \mathcal{R}_1 \cup \mathcal{R}_2$ (see Theorem S1). If $(x(t), y(t))$ enters $\mathcal{R}_1 \cup \mathcal{R}_2$ at a finite time $T > 0$, then $(x(t), y(t))$ enters either $\mathcal{R}_1 \setminus E_h$ or $\mathcal{R}_2 \setminus E_h$. Note that E_h cannot be reached in finite time due to uniqueness of solutions. This implies a sign switch in $z(t)$. It follows that $(x(t), y(t)) \in \mathcal{R}_1 \cup \mathcal{R}_2$ for all times $t \geq T$, and no further switch in the sign of $z(t)$ occurs.

Next, consider the set $\mathcal{M}_1$. Let $(x, y) \in \mathcal{M}_1$ such that $M_1(x, y) = 0$. It is $x \leq \frac{f}{d}$. If $x = \frac{f}{d}$, then it follows that $(x, y) = E_h$. Thus, consider $x < \frac{f}{d}$. It is

$$\begin{aligned} \frac{d}{dt} M_1(x,y) &= b(x - y + n) - \frac{(2b + d)}{2b}\Big(f - b(x - y + n) - dx\Big) \\ &\overset{(M_1=0)}{=} \frac{d}{4b}\Big(dx - f\Big) < 0. \end{aligned} \tag{1}$$

Then, positive invariance of $\mathcal{M}_1$ follows by positive invariance of $\mathcal{R}_1$. Next, $z(t)$ is shown to be monotonically decreasing. For that, let $(x^0, y^0) \in \mathcal{M}_1$. It follows that $M_1(x(t), y(t)) \leq 0$ for all $t \geq 0$. Therefore, it is

$$\begin{aligned} z'(t) &= f - 2bz(t) - dx(t) = f - 2bx(t) + 2by(t) - 2bn - dx(t) \\ &\leq f - 2bx(t) + 2b\left(\left(1 + \frac{d}{2b}\right)x(t) + n - \frac{f}{2b}\right) - 2bn - dx(t) = 0. \end{aligned}$$

This shows that $z'(t) \leq 0$ for all $t \geq 0$ in $\mathcal{M}_1$. Observe that if $M_1(x^0, y^0) < 0$, then $M_1(x(t), y(t)) < 0$ for all $t \geq 0$ by the strict inequality in (1). Consequently, $z'(t) < 0$ for all $t \geq 0$ in $\mathcal{M}_1$. The proof of the assertion for $\mathcal{M}_2$ is similar and left to the reader. □

Biological consequences

Theorem S2 allows studying the mode of decay of the surplus of PCs (outside or inside the niche) after an initial perturbation of PC homoeostasis. In particular, the dynamics of PCs after vaccination can be described. After an initial flow of PCs into the niche due to a high number of PCs outside the niche, the concomitant surplus of PCs inside the niche leads to PCs being expelled from the niche until PC homoeostasis is recovered. Such dynamics comes along with a switch in the sign of the function $z(t)$ (see Figure 3 in the main article).

Characterisation of the switching time

For a vaccine-induced perturbation of the healthy equilibrium at time T, the following result determines the switching time $\bar{t}$. It is $z(t) > 0$ for $T < t < \bar{t}$, and $z(t) \leq 0$ for $t > \bar{t}$.

Moreover, it provides an upper bound for the number of vaccine-specific PCs in the niche at the re-established healthy equilibrium.

Theorem S3. *Consider system* (SM1) *with initial conditions given by a vaccine-induced perturbation of the healthy equilibrium at* $T = 0$*. Then, it is* $y_v(t) \leq y_v^{upper}(\bar{t})$ *for all* $t \geq 0$*, where*

$$y_v^{upper}(t) := (1 - e^{-2bt})\frac{1}{2}x_v^0$$
$$\bar{t} := \frac{1}{\sqrt{4b^2 + d^2}} \ln\left(\frac{4b^2 + 2bd + d^2 + (2b + d)\sqrt{4b^2 + d^2}}{2bd}\right).$$

Proof. Since system (SM1) is a refinement of system (SM2), it follows that $z(0) > 0$. Observe that $(x^0, y^0) \notin \mathcal{R}_1 \cup \mathcal{R}_2$. Consequently, $z(t)$ is zero if either the solution has reached the equilibrium (which can only happen at infinite time due to the uniqueness of solutions), or the function is to change sign (in finite time). In any case, there is at most one (finite or infinite) time $\bar{t} > 0$ with $z(\bar{t}) = 0$. The proof consists of two steps: In the first step, it is used that $z(t) \geq 0$ for $0 \leq t \leq \bar{t}$. This enables the derivation of upper estimates for the solutions $y_0(t)$ and $y_v(t)$ of system (SM1), respectively. In the second step, evaluation of these estimates at the switching time $\bar{t}$ yields upper estimates for $y_0(t)$ and $y_v(t)$ for all $t \geq 0$, respectively. This is due to the following reasoning: On the one hand, it is $z(t) \geq 0$ for all $0 \leq t \leq \bar{t}$ implying $y_0'(t) \geq 0$ and $y_v'(t) \geq 0$ for all $0 \leq t \leq \bar{t}$. On the other hand, it is $z(t) \leq 0$ for all $t \geq \bar{t}$, and consequently $y_0'(t) \leq 0$ and $y_v'(t) \leq 0$ for all $t \geq \bar{t}$.

Step 1: Since

$$x_0'(t) = f - \beta_0(z(t))z(t) - dx_0(t) \leq f - dx_0(t)$$
$$x_v'(t) = -\beta_v(z(t))z(t) - dx_v(t) \leq -dx_v(t)$$

with $x_0(0) = x^{E_h} = \frac{f}{d}$ and $x_v(0) = x_v^0 > 0$, it follows that

$$x_0(t) \leq \frac{f}{d}, \quad x_v(t) \leq x_v^0.$$

Consequently, the following estimates hold true:

$$y_0'(t) = \beta_0(z(t))z(t) \leq bz(t) \leq u(y_0(t), y_v(t))$$
$$y_v'(t) = \beta_v(z(t))z(t) \leq bz(t) \leq u(y_0(t), y_v(t)),$$

where

$$u(y_0(t), y_v(t)) := b\left(\frac{f}{d} + x_v^0 - y_0(t) - y_v(t) + n\right).$$

Due to the comparison principle for systems of ODEs [22], it follows that

$$y_0(t) \leq y_0^{upper}(t)$$
$$y_v(t) \leq y_v^{upper}(t),$$

where $y_0^{upper}(t)$ and $y_v^{upper}(t)$ are solutions of the system

$$\frac{d}{dt}y_0^{upper}(t) = u(y_0^{upper}(t), y_v^{upper}(t)), \quad y_0^{upper}(0) = y_0(0) = \frac{f}{d} + n$$
$$\frac{d}{dt}y_v^{upper}(t) = u(y_0^{upper}(t), y_v^{upper}(t)), \quad y_v^{upper}(0) = y_v(0) = 0.$$

Solving this system gives

$$y_0^{upper}(t) = \frac{f}{d} + n + \frac{1}{2}x_v^0\left(1 - e^{-2bt}\right)$$
$$y_v^{upper}(t) = \left(1 - e^{-2bt}\right)\frac{1}{2}x_v^0.$$

Observe that the solutions are strictly positive for $t > 0$.

Step 2: The switching time $\bar{t}$ is calculated using explicit solution formulae for solutions $x(t)$ and $y(t)$ of system (SM2) for $z(t) \geq 0$,

$$x(t) = \frac{1}{2ds}e^{-\frac{1}{2}(2b+d+s)t}\left(2se^{\frac{1}{2}(2b+d+s)t}f + d(d+s)x_v^0 + d(-d+s)e^{st}x_v^0\right)$$
$$y(t) = \frac{f}{d} + \frac{bx_v^0}{s}\left(e^{st} - 1\right)e^{-\frac{1}{2}(2b+d+s)t} + n,$$

where $s = \sqrt{4b^2 + d^2}$ with initial conditions $x^0 = \frac{f}{d} + x_v^0$ and $y^0 = \frac{f}{d} + n$. They are derived via elementary calculation. This implies

$$z(t) = 0 \quad \Leftrightarrow \quad t = \bar{t} \ \text{ with } \ \bar{t} = \frac{1}{s}\ln\left(\frac{4b^2 + 2bd + d^2 + (2b+d)s}{2bd}\right)$$

Note that $\bar{t} > 0$ is well-defined. In particular, $\bar{t}$ only depends on the parameters b and d. As a consequence, a switch in the sign of $z(t)$ occurs at time $\bar{t}$. Combining steps 1 and 2 yields that $y_v^{upper}(\bar{t})$ provides an upper estimate for $y_v(t)$ for all $t \geq 0$. □

Biological consequences

Biologically, the switching time of the function $z(t)$ after a vaccine-induced perturbation of PC homoeostasis determines the time span of vaccine-specific PCs entering the niche and the onset of the time interval during which the PCs become out-competed. It solely depends on the rate of transition into the niche and the death rate of PCs outside the niche. As the upper bound for the number of vaccine-specific PCs at the recovered PC homoeostasis particularly depends on the number of vaccine-specific PCs arriving in the bone marrow, a larger number would permit more vaccine-specific PCs to establish themselves in the niche.

S4 Parameter estimation

Table S3. Estimates of undetermined parameters of the piecewise-exponential function $\tilde{g}(t)$ within the vaccination model (M) based on dataset (D1).

Parameter	Estimate	Confidence interval
a_1	$1.092 \cdot 10^{-4}$	$(1.069 \cdot 10^{-4}, 1.115 \cdot 10^{-4})$
b_1	2.996	$(2.9925, 2.9995)$
a_2	$a_1 e^{b1 \times 6} \approx 7000$ (fixed)	NA
b_2	-0.812903	$(-0.884207, -0.741599)$

Table S4. Estimates of undetermined parameters within the vaccination model (M) based on dataset (D2).

Parameter	Estimate	Confidence interval
α	$5.785 \cdot 10^{-5}$	$(5.076 \cdot 10^{-5}, 6.494 \cdot 10^{-5})$
ν	1.386	$(1.325, 1.448)$

For assessing practical identifiability of the parameters, additional estimations were performed using different optimisation methods and varying initialisations within a biologically reasonable range (see documentation of *Mathematica* (Version 9, *Wolfram Research*)). As a result, estimates and the widths of the corresponding confidence intervals did not significantly differ, indicating that the parameter values could uniquely be identified on the basis of the data.

Table S5. Estimates of undetermined parameters within the vaccination model (M) based on dataset (D2). Transition rate b is added to the set of estimated parameters, implying wider confidence intervals compared to Table S4.

Parameter	Estimate	Confidence interval
b	0.99	$(-0.441, 2.437)$
α	$5.46 \cdot 10^{-5}$	$(4.41 \cdot 10^{-5}, 6.52 \cdot 10^{-5})$
ν	0.476	$(1.058, 2.011)$

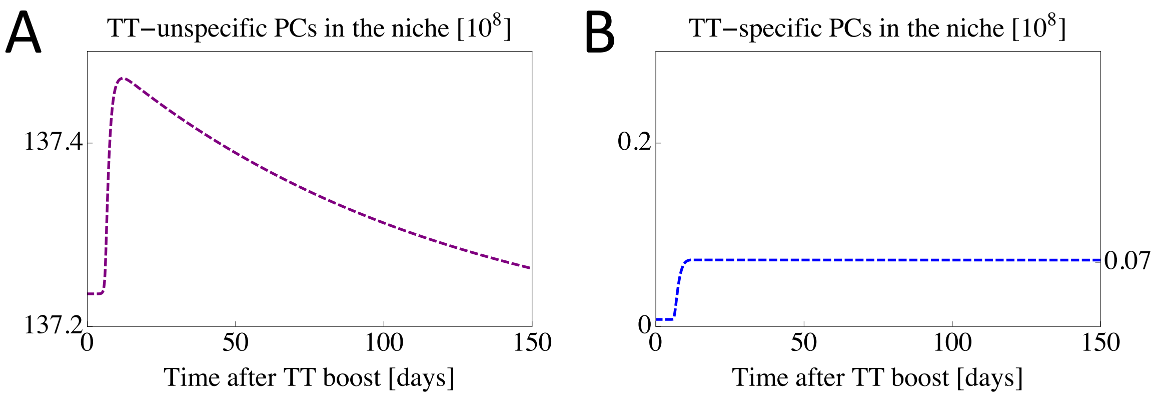

Figure S1. Out-competition of plasma cells (PCs) after tetanus-toxoid (TT) boost.

The obtained fit for the number of **(A)** TT-nonspecific PCs and **(B)** TT-specific PCs inside the niche is visualised. Both types of PCs enter the niche after TT boost, for the TT-nonspecific PCs explainable by their constant inflow. Competition mainly affects the TT-nonspecific PCs visualised by a declining number of TT-nonspecific PCs 12 days after the TT boost (i.e. the switching time). In contrast, the number of the TT-specific PCs inside the niche hardly changes. This is due to the model assumption stating that PCs leave the niche proportionally to their number within the niche. The latter is relatively low for the TT-specific PCs.

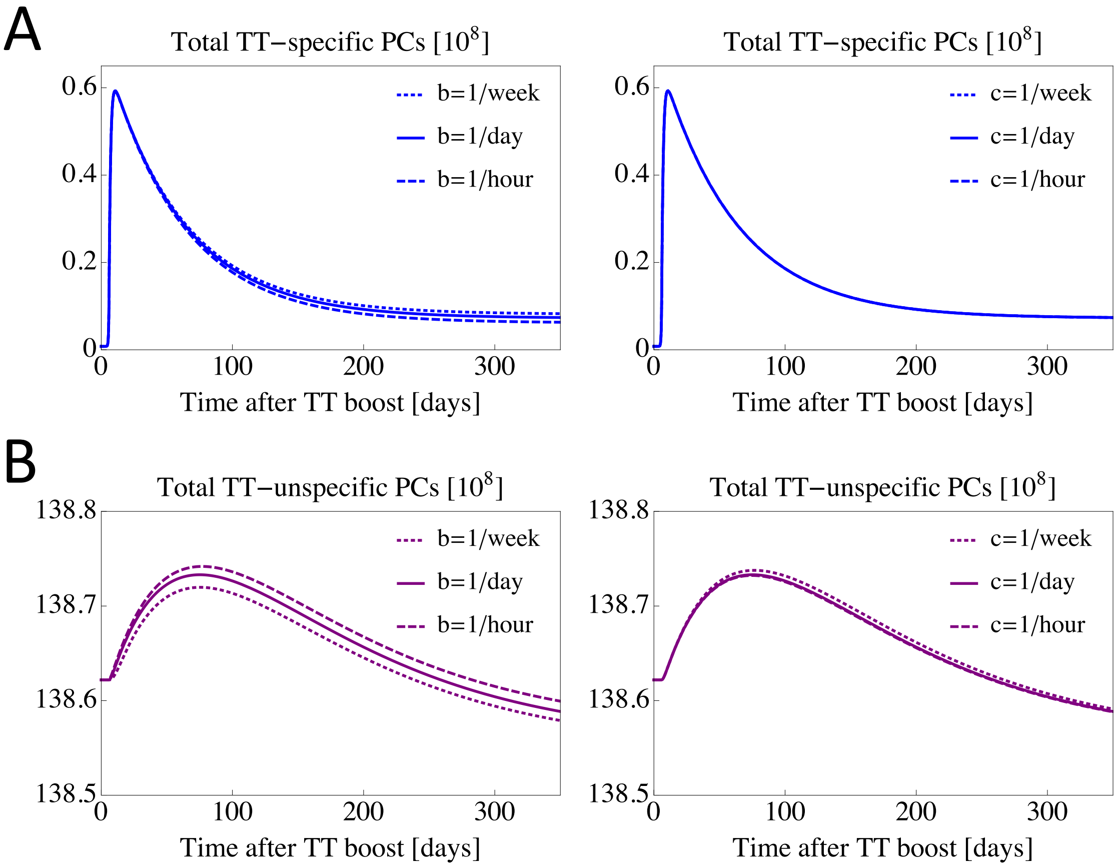

Figure S2. Variations in the total numbers of tetanus-toxoid (TT)-specific and nonspecific plasma cells (PCs) due to changes in the transition rates.
The obtained fit of the vaccination model (M) is analysed with respect to variations in the transition rates of PCs into and out of the niche. $b = 1/\text{day}$ (bold) is altered by increasing it to 1/hour (dashed), and by decreasing it to 1/week (dotted). Likewise, $c = 1/\text{day}$ (bold) is altered by increasing it to 1/hour (dashed), and by decreasing it to 1/week (dotted). **(A)** Total number of TT-specific PCs using the set of parameters related to the fit of the data and different values for the transition rates. Maximal relative deviations from the fit (bold) within the time range of the data are about 8% and 0.003% for variations in b and c, respectively. **(B)** Total number of TT-nonspecific PCs using the set of parameters related to the fit of the data and different values for the transition rates. Maximal relative deviations from the fit (bold) within the time range of the data are about 0.009% and 0.004% for variations in b and c, respectively.

Supplemental references

[1] Bernasconi NL, Traggiai E, Lanzavecchia A. Maintenance of serological memory by polyclonal activation of human memory B cells. Science. 2002;298(5601):2199–2202. doi:10.1126/science.1076071.

[2] Jolliff C, Cost K, Stivrins P, Grossman PP, Nolte CR, Franco S, Fijan K, Fletcher L, Shriner H. Reference intervals for serum IgG, IgA, IgM, C3, and C4 as determined by rate nephelometry. Clin Chem. 1982;28(1):126–128.

[3] Gonzalez-Quintela A, Alende R, Gude F, Campos J, Rey J, Meijide L, Fernandez-Merino C, Vidal C. Serum levels of immunoglobulins (IgG, IgA, IgM) in a general adult population and their relationship with alcohol consumption, smoking and common metabolic abnormalities. Clin Exp Immunol. 2008;151(1):42–50. doi:10.1111/j.1365-2249.2007.03545.x.

[4] Ellyard JI, Avery DT, Phan TG, Hare NJ, Hodgkin PD, Tangye SG. Antigen-selected, immunoglobulin-secreting cells persist in human spleen and bone marrow. Blood. 2004;103(10):3805–3812. doi:10.1182/blood-2003-09-3109.

[5] Radbruch A, Mühlinghaus G, Luger EO, Inamine A, Smith KG, Dörner T, Hiepe F. Competence and competition: The challenge of becoming a long-lived plasma cell. Nat Rev Immunol. 2006;6(10):741–750. doi:10.1038/nri1886.

[6] Harrison W. The total cellularity of the bone marrow in man. J Clin Pathol. 1962;15(3):254–259. doi:10.1136/jcp.15.3.254.

[7] Terstappen LW, Johnsen S, Segers-Nolten IM, Loken MR. Identification and characterization of plasma cells in normal human bone marrow by high-resolution flow cytometry. Blood. 1990;76(9):1739–1747.

[8] Sandkühler S, Gross E. Normal bone marrow total cell and differential values by quantitative analysis of particle smears. Blood. 1956;11(9):856–862.

[9] Wetzel G. Geschichtliches zur Bestimmung der Größe des Markorgans. Anat Embryol (Berl). 1927;82(1):70–72. doi:10.1007/bf02119510.

[10] Brekke OH, Sandlie I. Therapeutic antibodies for human diseases at the dawn of the twenty-first century. Nat Rev Drug Discovery. 2003;2(1):52–62. doi:10.1038/nrd984.

[11] Mankarious S, Lee M, Fischer S, Pyun K, Ochs H, Oxelius V, Wedgwood R. The half-lives of IgG subclasses and specific antibodies in patients with primary immunodeficiency who are receiving intravenously administered immunoglobulin. J Lab Clin Med. 1988;112(5):634–640.

[12] Morell A, Terry WD, Waldmann TA. Metabolic properties of IgG subclasses in man. J Clin Invest. 1970;49(4):673. doi:10.1172/jci106279.

[13] Jourdan M, Caraux A, De Vos J, Fiol G, Larroque M, Cognot C, Bret C, Duperray C, Hose D, Klein B. An in vitro model of differentiation of memory B cells into plasmablasts and plasma cells including detailed phenotypic and molecular characterization. Blood. 2009;114(25):5173–5181. doi:10.1182/blood-2009-07-235960.

[14] Fink K. Origin and function of circulating plasmablasts during acute viral infections. Front Immunol. 2012;3:78. doi:10.3389/fimmu.2012.00078.

[15] di Bernardo M, Budd C, Champneys AR, Kowalczyk P. Piecewise-smooth dynamical systems: Theory and applications. vol. 163 of Applied Mathematical Sciences. London: Springer; 2008.

[16] Hartman P. Ordinary Differential Equations. New York: John Wiley and Sons; 1964. doi:10.1137/1.9780898719222.

[17] Mohr M. Mathematical modelling of plasma cell dynamics in multiple myeloma. Dissertation; Heidelberg University; 2016.

[18] Wiggins S. Introduction to applied nonlinear dynamical systems and chaos. vol. 2 of Texts in Applied Mathematics. 2nd ed. New York: Springer; 2003. doi:10.1007/978-1-4757-4067-7.

[19] Guckenheimer J, Holmes P. Nonlinear oscillations, dynamical systems, and bifurcations of vector fields. vol. 42 of Applied Mathematical Sciences. New York: Springer; 1983. doi:10.1007/978-1-4612-1140-2.

[20] Carmona V, Freire E, Ponce E, Torres F. Invariant manifolds of periodic orbits for piecewise linear three-dimensional systems. IMA J Appl Math. 2004;69(1):71–91. doi:10.1093/imamat/69.1.71.

[21] Liberzon D. Switching in systems and control. Basel: Birkhäuser; 2012. doi:10.1007/978-1-4612-0017-8.

[22] Kirkilionis M, Walcher S. On comparison systems for ordinary differential equations. J Math Anal Appl. 2004;299(1):157–173. doi:10.1016/j.jmaa.2004.06.025.
